# Researches on the Possibility of Restoring Temporary Life to Individuals Dying of Disease

**Published:** 1859-07

**Authors:** E. Brownsequard


### ARTICLE XV.

*Researches on the Possibility of Restoring Temporary Life to Individuals Dying of Disease.* By DR. E. BROWN-SEQUARD. Translated from the Journal de la Physiologie de l'Homme et des Animaux.

FOR many years my researches on the Transfusion of Blood, begun in 1846, and continued to the present time almost without interruption, have frequently afforded me results showing with certainty that life may be restored, for a certain time, in mammalia dying of various diseases, and especially of peritonitis.

I shall merely report here a few of the principal facts observed, intending to publish the rest in an extended memoir on the transfusion of blood.

The successful experiments were made on animals who had ceased to breathe for a variable number of minutes (in one case, seventeen minutes,) and in whom all traces of voluntary movement and of sensation had disappeared. The sounds of the heart were still audible, in most of the cases, but there was no pulse in the arteries of the limbs, and often not even in the carotid ; in two cases the beating of the heart appeared to have ceased for several minutes. The death struggle had taken place, and the pupil was dilating or already dilated. In a word, death was imminent, and certainly no one could suppose that a spontaneous return to life was possible under such circumstances.

The diseases which had brought the animals to the verge of death, consisted chiefly of inflammation of the peritoneum or of the pleura, following injuries to these membranes, made in the course of the experiments upon the different abdominal viscera, or upon the vagi sympathetic or diaphragmatic nerves, in the thoracic cavity. In several cases the approaching death was owing to the extirpation of the supra-renal capsules, or of the kidneys.

In every case, without exception, asphyxia was the proximate cause of death. I do not intend to examine its mode of production, this question being too important to be treated incidentally, and I shall make it the subject of a series of papers on the causes of death in acute diseases in man and animals. I ought, however, to say here, that in a very large number of the cases of lesions of the abdominal viscera, in the experiments which form the basis of this paper, the asphyxia was principally the consequence of feebleness of the movements of the heart, the chief, but not the only cause of which, was an irritation of the ramifications of the great sympathetic nerve in the abdomen.

But, however produced, the asphyxia existed, and in endeavoring to restore life temporarily to the dying animals, it was consequently to be taken into consideration. I was thus led to try whether artificial respiration might have some influence, and I practiced it on eight or ten dying animals. In a few instances there was a slight increase in the strength of the movements of the heart, and one animal seemed to recover his consciousness for a few minutes. The case was very different when the insufflation was employed before the death struggle, but as that is foreign to the subject, I will only remark that we may generally defer the last agony from one to several hours by means of pulmonary insufflation.

This means being inadequate to restore life to animals dying from febrile diseases, I endeavored to discover whether by employing others I might not succeed better. I would say to those who imagine that galvanism may be useful as a means of resuscitating the asphyxiated, that I did not employ it once, because I know that the surest way to destroy the remnant of life in the dying is to subject their nerves and their muscles to the stimulating and exhausting influence of galvanism. (See *Lois des phénomenes dynamiques de l'économie animale*, No.1., pp. 7-10 of the *Journal de Physiologie.*) I thought then of trying the transfusion of blood, not with the insane idea of the early transfusors, who thought it possible to cure the gravest diseases by substituting fresh blood for diseased blood, but with the desire of ascertaining the effect of this operation on an organism scarcely living, and necessarily condemned to a speedy death.

I performed the operation in different ways, but, on account of my limited space, I shall only describe, in reporting the following case, the method which succeeded best, and I shall afterward add a general description of some modifications of this proceeding.

*Experiment.*—In October, 1851, a dog, whose grand sympathetic nerve I had tied in the abdomen, was attacked with peritonitis, and after two or three days sickness, exhibited the signs of approaching death, so well known to vivisectors. The voluntary movements had already ceased for some time, (I did not note the exact time.) Sensation and reflex action were suspended in every part, even in the eyes. The last respiratory movements (those of the jaws and nostrils) had stopped. The death struggles (very feeble in this case) in the limbs, the face and the eyes, consisted only of trembling, limited to a very few muscles. The animal had expelled fæces and urine; the pupil was dilating, and the beating of the heart could no longer be felt. The only sign of life still remaining consisted in an indistinct sound unlike the sounds of the heart, which was heard about eight times during the twelve seconds preceding the transfusion of blood from another dog, which was performed in the following manner. A silver tube of a T-shape, was attached by the branch corresponding to the cross of the T to the interior of the right carotid of the dying dog, while the free extremity of the other branch, slightly curved, was introduced into the left carotid of another dog, whose head and body were firmly secured to a table. The blood of the healthy dog immediately circulated in two opposite directions, toward the head, and toward the heart, in the carotid of the dying animal. At the same time, the left jugular, and one of the femoral veins of the same animal were opened. At first these veins did not bleed, but almost immediately blood poured from the jugular, and after twenty or thirty seconds, some came from the femoral. During the first fifteen seconds by compressing the carotid which gave issue to the transfused blood, I diminished the quantity of this liquid, and at the end of two minutes the operation was terminated, and ligatures were placed upon the carotid and vein of the dying animal. The jugular vein was left open for four or five minutes, during which the pulsations of the heart began to be perceived. In proportion as the blood flowed from the jugular, the pulse returned. I then had recourse to artificial respiration. As I had but a single assistant, the disturbance caused by the experiment prevented me from counting the pulse during the first twelve minutes. When it was counted, it was very feeble, beating sixty-four times per minute. The insufflation was continued almost uninterruptedly during half an hour. There was sensation in the cornea from the eighth minute after the commencement of the insufflation; a few minutes later, inspiratory efforts took place. After twenty minutes, the animal made voluntary movements, and when the insufflation was stopped, respiration took place, which was quick, but without much strength. The restoration to life, however, was complete as to the existence of all the principal functions of animal and organic life. The animal, although feeble, raised himself on his fore legs, and wagged his tail when caressed. The small and feeble pulse was rapid (110 to 120; several hours afterward, 80.) After remaining four or five hours in this state, the animal sank again, and died (I was about to say *died again*) eleven and a half hours after the transfusion.

I shall add only a few remarks to the details of this experiment. I did not weigh the dogs who were the subjects of it, and I do not know the amount of blood which was transfused, nor how much was lost by the dying dog. But I observed that the animal which furnished the blood was small, and that the other was large, so that, judging from these facts, from the duration of the operation, and from several other circumstances, I have reason to believe that the dying animal did not receive more blood than he lost. Subsequent experiments, however, have shown me that the quantity of blood transfused was too large, and that it is probable the resuscitation would have been more rapid if this quantity had been less.

In this experiment there were several causes for the return to life: 1, the passage of normal arterial blood through the coronary arteries, giving more irritability to the muscular fibres of the heart, and consequently rendering them more capable of obeying the stimulus (whatever it be) which causes them to contract rythmically; 2, the passage of normal arterial blood in the cephalic arteries; 3, the partial substitution of normal blood for blood altered by an inflammatory disease, and by the asphyxia existing during the last struggles; 4, the insufflation of the lungs; 5, the relief to the distended right cavities of the heart, by the bleeding from the jugular vein.

Of these five causes of the temporary restoration to life, there is one, artificial respiration, which, as I have already said, is incapable of itself, of producing this result. As to the others, I may say that they are nearly as inefficacious as insufflation, if employed alone. Thus, the injection of arterial blood toward the heart by the carotid, in dying animals, is capable only of increasing for a time the force and rapidity of the movements of this organ, but without restoring the circulation; so, also, the transfusion of blood by a vein only hastens the complete stoppage of the motion of the heart, if it is so performed that the right ventricle cannot, from an opening in the jugular, relieve itself of the distension which prevents it from contracting in asphyxia. Likewise transfusion by the carotid toward the brain only adds to the difficulty of contraction of the right ventricle; and, finally, opening the jugular, alone, gives more freedom of action to the right side of the heart, but only for a very short time.

A long time ago, M. Ségalas, and, after him, J. Reid, Dr. Cormack, Dr. H. Lonsdale, and finally Dr. Struthers, ascertained that in asphyxia from hanging, from blows on the head, and in certain cases of poisoning, the movements of the heart could be rendered more active, or could be restored if they had ceased, by bleeding from the jugular, and thus relieving the distension of the right cavities of the heart. This excellent method, if employed alone on an animal dying of an inflammatory disease, is absolutely inefficacious. Even by combining with it pulmonary insufflation, I have never succeeded in restoring the circulation, and still less the respiration and the functions of animal life; the only result has been an increase in the activity of the movements of the heart. But in the cases in which I emt ployed both these means at the very beginning of the agony, and still better, before this was manifested by convulsions, I have frequently seen the respiration and circulation restored for one or two hours, and sometimes there was a momentary return of the functions of animal life.

In a number of cases I tried transfusion by the carotid in two opposite directions, taking care to keep the jugular open during, and a little after, the transfusion, and without performing artificial respiration. The resuscitation in these cases was more rare than when insufflation was practised, but it took place in several animals, who lived one, two or three hours.

Out of the number of trials which I made, I cannot tell what is the actual proportion of those animals who were restored to life for a certain time, nor what was the average duration of this new life in the dying animals upon whom I performed transfusion, insufflation and bleeding from the jugular, because I have not at hand all my records of experiments ; but from such notes as I have before me, I find that in eleven experiments, made on fullgrown dogs, cats and rabbits, nearly all dying of peritonitis, four animals were quite restored to life, during two, three and four hours, (one dog, one cat, and two rabbits,) and that three others recovered during one or two hours, their circulation, respiration and reflex action, without restoration of voluntary motion or sensation, while in the four others there was no result, except a slight augmentation in the movements of the heart.

Having of late years, after numerous investigations on the transfusion of blood, discovered that the blood employed need not be warm, and need not contain fibrine,* I have ceased to employ a healthy animal to effect the transfusion.

* I have recently found, however, that defibrinated blood offers this danger, that when mingled with the blood of the animal into whom it is transfused, it sometimes causes a sudden coagulation ; but it is probable that by adding a thousandth or fifteen hundredth part of caustic ammonia to the defibrinated blood, the only danger to its use, so far as I know, may be avoided.

After drawing blood from the jugular, and having commenced artificial respiration, I inject, at several intervals, and very slowly, a quantity of blood rather less than the animal has lost. I direct the injection alternately toward the heart, and toward the head, in order to act on the brain, with a view to restore the respiration, and on the muscular fibres of the heart to increase their irritability.

Can we derive from these experiments any consequences relative to the combined employment of transfusion, insufflation and bleeding from the jugular, in individuals of the human species, dying of inflammatory or other diseases? It would evidently be useless, if not cruel, in the great majority of cases, to snatch from death, for a time necessarily very brief, one of our fellow beings condemned to death by irreparable structural lesions. But cases might occur in which it would be important that intelligence, speech, the senses and voluntary motion should be restored to the dying. Now the facts stated in this paper, showing that *all the functions of animal life may be restored for several hours in animals in whom the final convulsions have given place almost completely to death*, render it extremely probable that the intellectual faculties, the senses, the speech, &c., might be restored for several hours in patients who have lost their faculties, and in whom the death struggle had commenced. The success of these operations is the more probable, since we need not wait, as I have done, in experimenting on animals, until the death agony has made considerable progress, and still less, until it has almost completely terminated.

